# A Deadly Disease of Our Own Times

**Published:** 1895-03-09

**Authors:** 


### The Hospital

AN INSTITUTIONAL JOURNAL OF

The Medical Sciences and Hospital Administration,

WITH

AN EXTRA NURSING SUPPLEMENT.

VOL. XVII.—No. 441.] [MARCH 9, 1895.

### A DEADLY DISEASE OF OUR OWN TIMES.

THERE have been competent critics who have declared that the "Diary of a Late Physician," which was a work of fiction, consisted of mere tame commonplace compared with the actual facts which would be revealed by the carefully-kept diary of any modern physician whose life-work has lain at one of the larger centres of civilisation. Here is a sentence, which if taken from the pages of a novel would be considered to be more than bordering on the sensational: "One terrible form of brain disease, with mental symptoms, is certainly increasing. . . . That malady may be described as a breakdown of the great centres of mind and motion in the brain: it always goes on from bad to worse till it renders its victim utterly helpless in mind and body, and kills him in a few years. No cure, and scarcely any mitigation of this latter-day curse, has yet been devised. It is a disease of cities, of restless lives, of active brains in their prime; sometimes of dissipation and debauchery, of life at high pressure commonly." These are not the words of a novelist, resolved upon producing a sensation at any cost. They are taken from that least sensational of all the forms of printed matter, an annual report; and that the annual report of a sober scientist who speaks only what he knows and testifies only what he has seen. It is Dr. T. S. Clouston, Superintendent of the Morningside Asylum, and one of the most widely known and trusted of mental physicians, who thus, with all sobriety and the fullest possible sense of responsibility, utters his prophesy to his fellow-countrymen.

The old world gave attention to its seers and thinkers, though their habit was to deal largely with abstractions and general propositions. The modern world has no time to consider what its seers and thinkers may mean, although they deal with the grimmest and deadliest of physical realities and tragedies which are being enacted every day on the great world's stage before our very eyes. Lord Randolph Churchill, in the prime of his manhood and powers, died of general paralysis, yet that object lesson was not even a nine days' talk. It is not the illiterate and the vulgar only who have not eyes for the revelations of science. The readers of newspapers—the readers and the writers—are equally blind.

Dr. Clouston, although he is the superintendent of but one asylum, has taken the trouble to collate the facts of his own institution with the similar facts of other like institutions in England, Scotland, and Ireland. He tells us that last year Scotland, with her small population of four million souls, furnished 150 cases of this dread and incurable disease—general paralysis—to her asylums, besides the many cases which must have occurred in private practice. England, with her larger population and still greater strenuousness of city life and activity, gave the startling total of 1,400 cases to her asylums. Ireland, with a population equal to that of Scotland, only sent 52 cases. What do these facts mean? They have a terrible significance to him who knows how to look beneath the surface until he brings into view the causes in full operation. What are the causes? Is it some new development of civilisation which has brought new causes into play? By no means. It is the intensification of old factors, of factors which constitute parts of the essential nature of all mankind; causes which must always exist, and which will go on becoming yet more intense and more deadly if we have not intellectual capacity and resolution enough to put a firm checking hand upon them. The most frequent of all causes, Dr. Clouston tells us, is "alcoholic excess"; next come "worry and trouble," then "adverse circumstances," then "over-work and strain," and finally "love and religion."

There is no cure, we are told, for this deadly disease of general paralysis: no cure although it attacks some of the most powerful and versatile intellects among the statesmen, scientists, men of affairs, novelists, and newspaper writers of our strenuous period. No cure, perhaps, but a preventive certainly there is. But who among us has mind enough to adopt the systematic use of preventives? It requires a very far-seeing man, indeed, to look weeks, months, and years ahead, and to see the outlines of general paralysis as a shadowy but grim ghost waiting to usher him into the terrible shades of mental and bodily night. But the outlines, and the shadowy ghost, and the solid reality are all there waiting; and waiting for some who now would overwhelm us with their contemptuous scorn if we did but so much as hint to them the bare possibility of such an ending to their brilliant and all-engrossing careers. That is the pity of it.

How may the brain worker prevent the approach of general paralysis? By one way, and one only. He must compel himself to keep a due balance between his physical income and his mental expenditure. There is no other way. At bottom it is a pure matter of physique; of mental and physical pounds, shillings, and pence. Food, sleep, exercise, rest; these are the indispensables and essentials of continuous mental work. When they begin to fail, or when any one of them begins to fail, then let us take out our field glasses and look away into the far distance for the shadow of general paralysis. When work interferes with food, sleep, exercise, or rest, then the work must be curtailed or changed. Must be; there is absolutely nothing else for it. These are the hard, unyielding, mathematical certainties of modern physiology. Let the man of literary and other strenuous forms of brain work despise these certainties if he will. If he does so, then whoever may escape a day of physiological judgment and final account, he will not.

